# Antinociceptive tolerance to NSAIDs microinjected into dorsal hippocampus

**DOI:** 10.1186/2050-6511-15-10

**Published:** 2014-02-28

**Authors:** Gulnazi Gurtskaia, Nana Tsiklauri, Ivliane Nozadze, Marina Nebieridze, Merab G Tsagareli

**Affiliations:** 1 Dept of Neurophysiology, Ivane Beritashvili Center for Experimental Biomedicine, Gotua Street 14, Tbilisi 0160, Georgia

**Keywords:** Antinociception, Endogenous opioids, Hot plate test, Non-opioid tolerance, NSAIDs, Tail-flick reflex

## Abstract

**Background:**

Pain is characterized as a complex experience, dependent not only on the regulation of nociceptive sensory systems, but also on the activation of mechanisms that control emotional processes in limbic brain areas such as the amygdala and the hippocampus. Several lines of investigations have shown that in some brain areas, particularly the midbrain periaqueductal gray matter, rostral ventro-medial medulla, central nucleus of amygdala and nucleus raphe magnus, microinjections of non-steroidal anti-inflammatory drugs (NSAIDs) induce antinociception with distinct development of tolerance. The present study was designed to examine whether microinjection of NSAIDs, clodifen, ketorolac and xefocam into the dorsal hippocampus (DH) leads to the development of antinociceptive tolerance in male rats.

**Methods:**

The experiments were carried out on experimental and control (with saline) white male rats. Animals were implanted with a guide cannula in the DH and tested for antinociception following microinjection of NSAIDs into the DH in the tail-flick (TF) and hot plate (HP) tests. Repeated measures of analysis of variance with post-hoc Tukey-Kramer multiple comparison tests were used for statistical evaluations.

**Results:**

We found that microinjection of these NSAIDs into the DH induces antinociception as revealed by a latency increase in the TF and HP tests compared to controls treated with saline into the DH. Subsequent tests on days 2 and 3, however, showed that the antinociceptive effect of NSAIDs progressively decreased, suggesting tolerance developed to this effect of NSAIDs. Both pretreatment and post-treatment with the opioid antagonist naloxone into the DH significantly reduced the antinociceptive effect of NSAIDs in both pain models.

**Conclusions:**

Our results indicate that microinjection of NSAIDs into the DH induces antinociception which is mediated via the opioid system and exhibits tolerance.

## Background

Emotional distress is an intrinsic and the most undesirable feature of painful states. Pain is characterized as a complex experience, dependent not only on the regulation of nociceptive sensory systems, but also on the activation of mechanisms that control emotional processes in limbic brain areas such as the amygdala and the hippocampus [1]. The involvement of the hippocampal formation (HF) in nociception has been suggested in several studies [2-4]. Just recently, some abnormalities in hippocampal functioning with persistent pain have been shown [5]. Particularly, mice with spared nerve injury (SNI) neuropathic pain were unable to extinguish contextual fear and showed increased anxiety-like behavior. Additionally, mice with SNI compared with sham animals exhibited hippocampal reduced extracellular signal-regulated kinase expression and phosphorylation, decreased neurogenesis, and altered short-term synaptic plasticity [5].

Furthermore, morphine microinjections in the dorsal hippocampus (DH) produced antinociceptive effects in the formalin-induced orofacial pain model in rats [6]. Recent evidence suggests the participation of cholinergic, opioidergic and GABA-ergic systems of the DH in the modulation of nociception in guinea pigs [2]. Moreover, opioid peptides are important modulators of information processing in the hippocampus. When activated, opioid receptors play a key role in central pain modulation mechanisms, and the HF is a structure that expresses significant densities of this kind of receptors [7,8]. In addition, the hippocampus is anatomically connected to components of the pain neuromatrix, including the amygdala and the descending pain pathway with the periaqueductal gray (PAG) – the rostral ventromedial (RVM) region of medulla [9]. However, specific neural substrates and circuitry mediating opioid-induced hippocampal antinociception are unknown.

We have recently shown that in the PAG, the central nucleus of amygdala (CeA), and the nucleus raphe magnus (NRM), microinjection of non-steroidal anti-inflammatory drugs (NSAIDs) induces antinociception with some effects of tolerance and cross-tolerance to morphine [10-14]. These findings strongly support the suggestion of endogenous opioid involvement in NSAIDs antinociception and tolerance in the descending pain-control system [15-19]. However, involvement of NSAIDs antinociception in the HF is still a matter of controversy. For example, indomethacin did not protect against significant pain-induced down-regulation of neurokinin-1 (NK-1) and brain derived-neurotropic factor (BDNF) receptor genes in the hippocampus, suggesting that although analgesic drug treatment reduces nociceptive sensory activation in the spinal cord, it is insufficient to prevent the impact of pain on the hippocampus [20].

In this study, we have examined whether microinjection of the widely used NSAIDs diclofenac, ketorolac, and xefocam into the DH induces antinociception and whether this action is mediated via the endogenous opioid system.

## Methods

### Animals

The experiments were carried out on male Wistar rats, 200-250 g in body weight, bred at Beritashvili Center for Experimental Biomedicine. The experimental protocol was approved by the local Bioethic Committee of the Center. Every effort was made to minimize both the number of animals used and their suffering. Guidelines of the International Association for the Study of Pain regarding animal experimentation were followed throughout [21].

### Surgical procedures

Under anesthesia with thiopental (55 mg/kg, i.p. “Kievmed”, Ukraine), a 12 mm-long stainless steel guide cannula (Small Parts, Inc, USA) was stereotaxically implanted into the DH bilaterally (AP:-4.3; L:±2.5; H:2.8) according to the coordinates in the atlas of Paxinos and Watson [22] siting the tip 2 mm above the DH. Guides were anchored to the cranium by dental cement. The guide cannula was plugged with a stainless steel stylet. Thereafter, the rats were handled every day for 3 days for 15 min. During this time, the stylet was removed and 14 mm-long stainless steel microinjection cannula was inserted into the guide cannula to reach the DH, but no drug was injected. This helped to habituate the rats to the injection procedure and to reduce artifacts arising from mechanical manipulation during the test days. Five days after surgery a microinjection cannula, attached to a 50-μl Hamilton syringe (Hamilton, Inc, USA), was introduced through the guide cannula, and the drug was microinjected while the rat was gently restrained.

### Drugs

Clodifen (diclofenac sodium, 75 μg/0.5 μl, RotexMedica, Germany), ketorolac (ketorolac tromethamine, 90 μg/0.5 μl, Zee Drugs, India) or xefocam (lornoxicam, 12 μg/0.5 μl, Nycomed, Austria) were injected through the microinjection cannula; the guide cannula was then plugged with a stainless steel stylet. Saline was injected in the same volume (0.5 μl, GalichPharm, Ukraine) and manner in a separate group of rats for controls. Solutions were microinjected in about 10 seconds.

### Behavioral testing

Twenty minutes post microinjection, i.e. 10-min before the peak of the drugs’ effect is normally reached, animals were tested for antinociception using the tail-flick (TF) and hot plate (HP) tests. For the TF test, the distal part of the tail was stimulated with a light beam and the latency measured until the tail was reflexively flicked away from the beam (IITC #33, IITC Life Science, Inc., Woodland Hills, CA, USA). For the HP test, the rat was placed on a 55ºC hot plate and the latency to the first hindpaw licking or jumping was measured (IITC #39). The cut-off time was 20 s for both TF and HP latencies. Each rat was tested with both tail flick (TF) and hot plate (HP) in the same session. A similar procedure was followed for the repeated microinjection of clodifen, ketorolac, xefocam or saline for four consecutive days.

In control experiments, saline microinjections into the DH was followed by a non-selective opioid receptor antagonist naloxone (0.5 μl, GalichPharm, Ukraine) and tested for TF and HP latencies for the three consecutive days.

In the second set of experiments, twenty minutes after NSAIDs administration we tested on whether post-treatment with a non-selective opioid receptor antagonist naloxone in the DH diminishes NSAID-induced antinociception on the 1st, 2nd and 3rd experimental days. In the third set of experiments, rats pretreated with the same dose of naloxone in the DH were followed by TF and HP tests. 10 min after rats were treated with NSAIDs in the same dose as in the first and second set of experiments and were then tested again. Different animal groups were used for experiments 1, 2 and 3. The number of rats in each group was five or six.

### Histology

**Figure 1:**
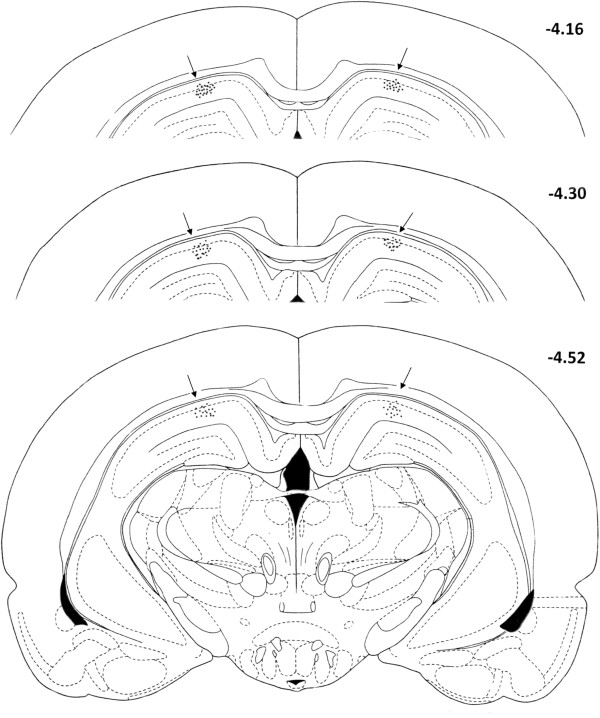
**Serial coronal sections of the rat brain showing placement of microinjections bilaterally in the DH (black arrows).** The numbers to the right of sections represent millimeters relative to bregma, adapted from the Paxinos and Watson (1997) stereotaxic atlas [22].

At the end of each set of experiments, the microinjection sites were marked with 2 μl of saturated solution of Pontamine Sky Blue (Sigma Chemical, Co.) and the animal was euthanized with an overdose of ester. After fixation by immersion in 10% formalin, the brain was sectioned and counterstained with Cresyl Violet. The microinjection sites were histologically verified and plotted according to Paxinos and Watson (1997) stereotaxic atlas coordinates [22]. Representative microinjection sites are shown in Figure 1.

### Statistical analysis

All data are presented as mean±S.E.M. Repeated measures of analysis of variance (ANOVA) with *post-hoc* Tukey-Kramer multiple comparison test were used for statistical comparisons between treated and saline groups, and treated and naloxone groups, respectively. The Kolmogorov–Smirnov test was applied to verify normality. The statistical software utilized was InStat 3.05 (GraphPad Software, USA). Statistical significance between vehicle control and treated groups, and naloxone and treated groups of rats was acknowledged if P < 0.05.

## Results

**Figure 2:**
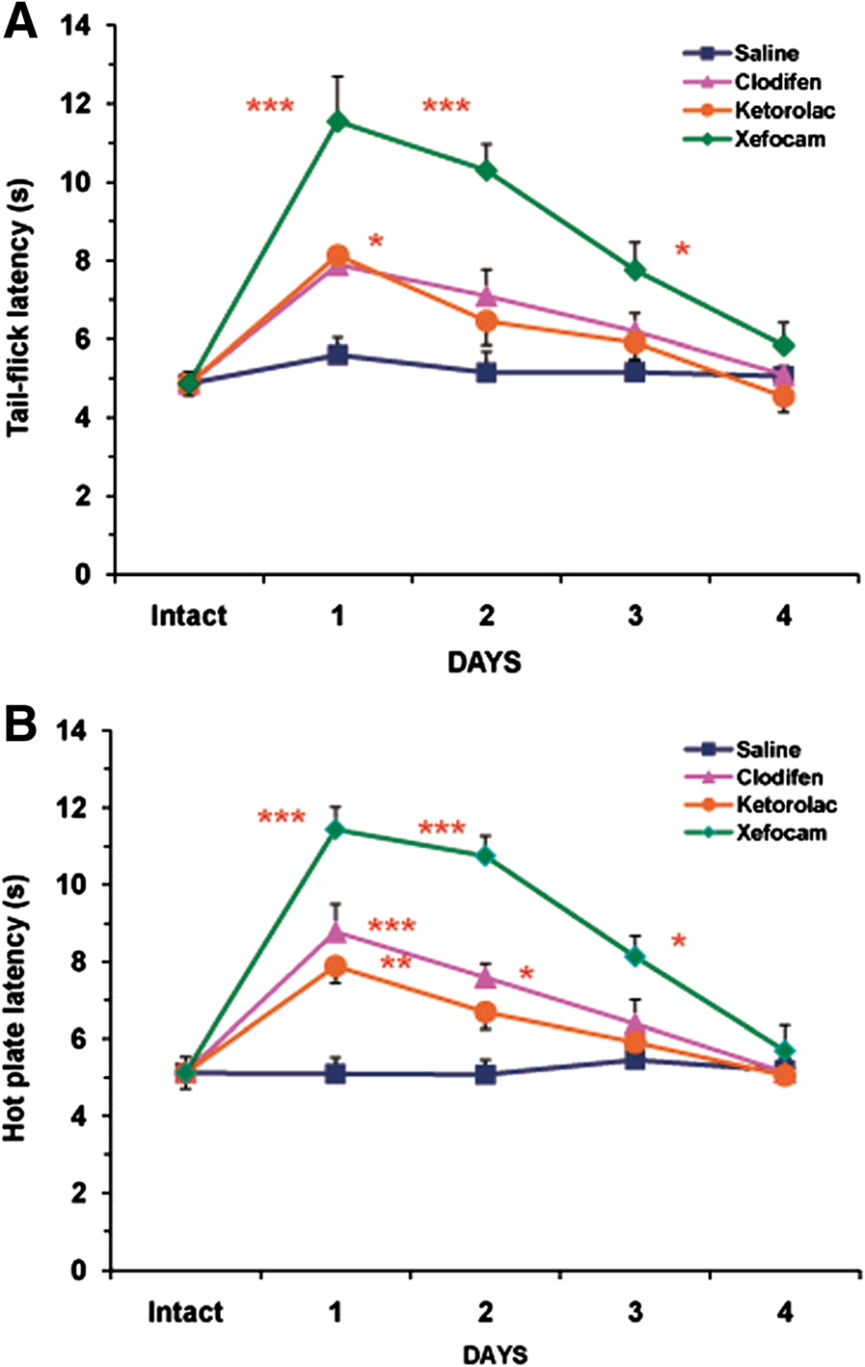
**Microinjections of NSAIDs into the DH for four consecutive days result in a progressive decrease in TF (A) and HP (B) latencies as compared to vehicle saline control.** The number of rats in the control group N = 16/group, in the treated groups for clodifen N = 5/group, for ketorolac N = 6/group, and for xefocam N = 5/group, respectively. *- P < 0.05, **- P < 0.01, ***- P < 0.001.

We found that microinjection of NSAIDs into the DH produced antinociception as revealed by a latency increase in TF and HP compared to the baseline control of intact rats and a control group with saline microinjected into the same site as well. The TF latency significantly increased for clodifen [ANOVA: F(4, 16) = 20.189, P < 0.0001], ketorolac [ANOVA: F(4,20) = 22.314, P < 0.0001], and xefocam [ANOVA: F(4,16) = 32.42, P < 0.0001]. We found similar significant differences in the HP latencies for clodifen [ANOVA: F(4,16) = 21.53, P < 0.0001], for ketorolac [ANOVA: F(4,20) = 17.764, P < 0.0001], and for xefocam [ANOVA: F(4,16) = 39.463, P < 0.0001], respectively. Subsequent NSAIDs microinjections caused progressively less antinociception, so by day 4 there was no effect, similar to saline microinjections for both the TF and the HP tests (Figure 2).

**Figure 3:**
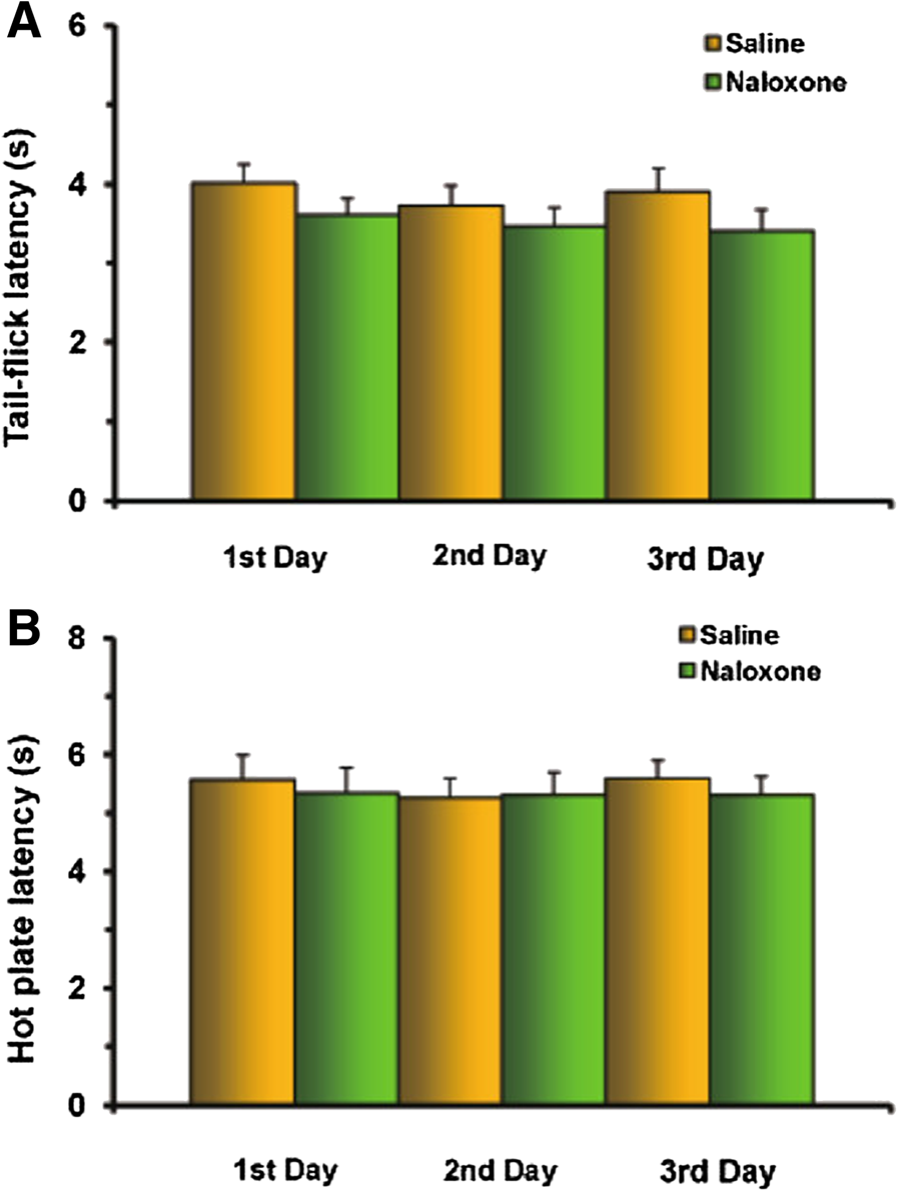
**Control experiments of post-treatment with naloxone after microinjection of saline into the DH does not significantly change TF (A) and HP (B) latencies either for the first or second and third days (P > 0.05).** Number of rats N = 5/group.

Control testing with saline microinjections into the DH followed by a non-selective opioid receptor antagonist naloxone statistically did not change the latency to respond in the TF [ANOVA: F(5,24) = 0.8914, P = 0.5024, not significant] and HP [ANOVA: F(5,24) = 0.1463, P = 0.9792, not significant] tests respectively for the first, second and third days (P > 0.05) (Figure 3A, B).

**Figure 4:**
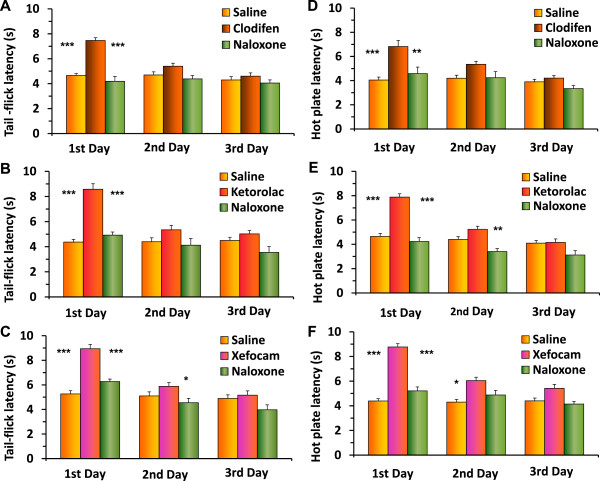
**Post treatment with naloxone after microinjections of NSAIDs into the DH results in a significant decrease in TF latency for the first day for clodifen (A), ketorolac (B), and xefocam (C), respectively (P < 0.001).** On the second and third days, there are generally trend effects for all three non-opioid analgesics. The same decreases of latencies are observed in HP test for clodifen **(D)** (P < 0.01), ketorolac **(E)** (P < 0.001), and xefocam **(F)** (P < 0.001), respectively. Number of rats N = 5/groups. *- P < 0.05, **- P < 0.01, ***- P < 0.001.

In the second set of experiments, we tested if post-treatment with the non-selective opioid receptor antagonist naloxone in the DH diminishes NSAID-induced antinociception at the first, second and third experimental days. Twenty minutes after NSAID administration, microinjection of naloxone in the DH significantly decreased antinociceptive effects of these drugs at the first day in the TF for clodifen [ANOVA: F(5,20) = 26.906, P < 0.0001], (t = 13.161, P < 0.001) (Figure 4A), for ketorolac [ANOVA: F(5,20) = 24.701, P < 0.0001], (t = 10.691, P < 0.001) (Figure 4B), and for xefocam [ANOVA: F(5,20) = 22.412, P < 0.0001], (t = 9.745, P < 0.001) (Figure 4C). At the second and third experimental days, naloxone showed generally trend effects (Figure 4), except for xefocam at the second experimental day where the difference between xefocam and naloxone injected groups is significant (P < 0.05) (Figure 4C).

The same effects we discovered in the HP test for clodifen [ANOVA: F(5,20) = 11.341, P < 0.0001], (t = 6.679, P < 0.01) (Figure 4D), for ketorolac [ANOVA: F(5,20) = 33.093, P < 0.0001], (t = 12.141, P < 0.001) (Figure 4E), and for xefocam [ANOVA: F(5,20) = 35.494, P < 0.0001], (t = 13.068, P < 0.001) (Figure 4F, C). At the second and third experimental days, naloxone showed generally trend effects (Figure 4), except for ketorolac in the second experimental day where the difference between ketorolac and naloxone injected groups is significant (P < 0.01) (Figure 4E).

**Figure 5:**
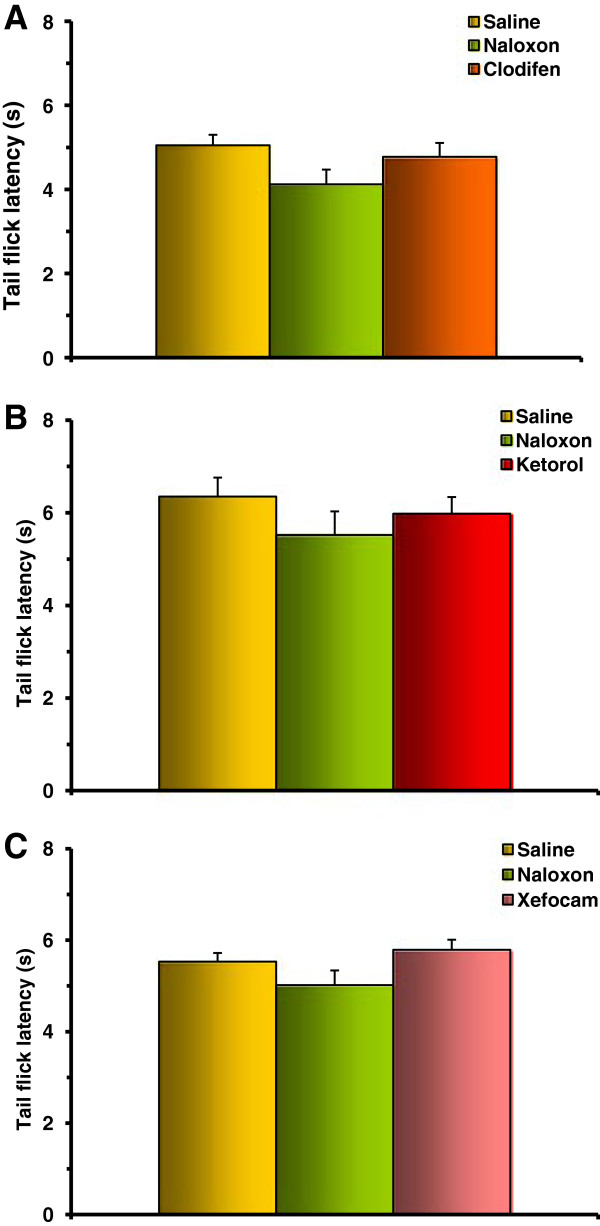
**Experiments of pretreatment with naloxone before microinjections of NSAIDs into the DH results in prevention of NSAID-induced antinociception in TF latency for clodifen (A), ketorolac (B), and xefocam (C), respectively.** Number of rats N = 5/groups.

**Figure 6:**
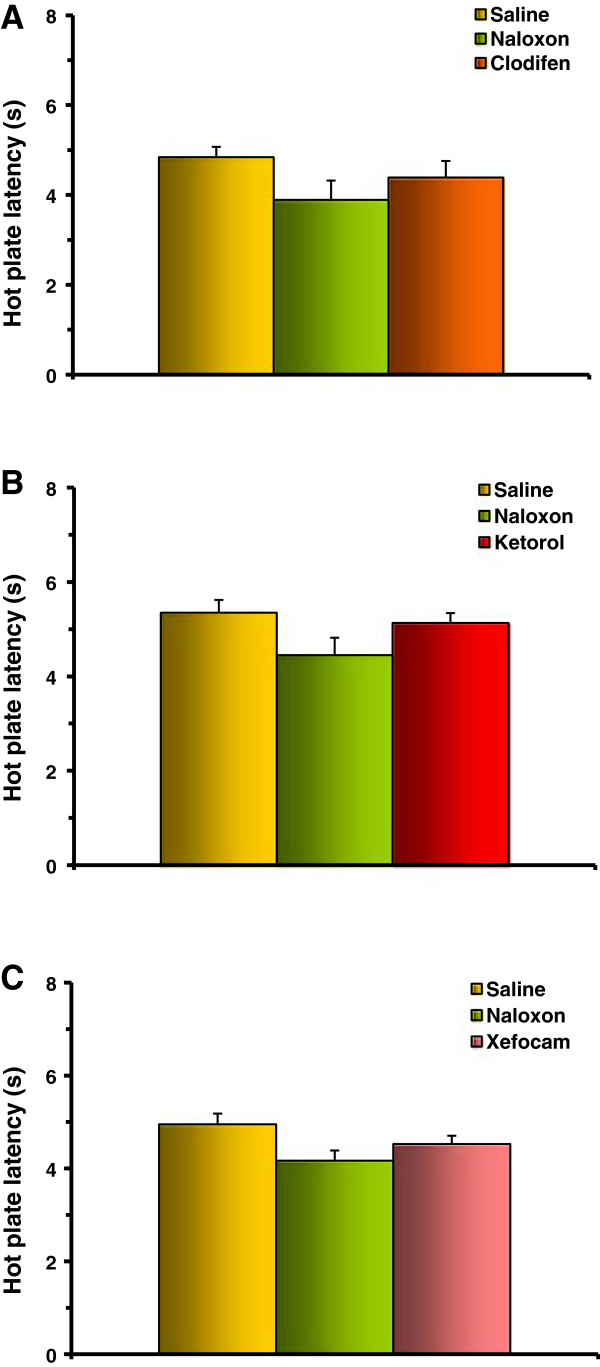
**Experiments of pretreatment with naloxone before microinjections of NSAIDs into the DH results in prevention of NSAID-induced antinociception in HP latency for clodifen (A), ketorolac (B), and xefocam (C), respectively.** Number of rats N = 5/groups.

In the third set of experiments, we tested if pretreatment with naloxone prevents antinociception induced by NSAID microinjected into the DH. Pretreatment with naloxone completely prevented the analgesic effects of clodifen (Figure 5A), ketorolac (Figure 5B), and xefocam (Figure 5C) in the TF test. The differences between NSAIDs injected and naloxone injected groups are not significant (Figure 5). The same results are in the HP test for clodifen (Figure 6A), ketorolac (Figure 6B), and xefocam (Figure 6C), respectively.

## Discussion

The present results demonstrate that microinjections of the NSAIDs, clodifen, ketorolac and xefocam into the DH induce antinociception. This confirms our and other colleagues previous results with systemic, intraperitoneal administration of NSAIDs [23,24], and results using microinjection of the same NSAIDs into the PAG [15,16]. In the other experiments in rats, responses of spinal dorsal horn wide-dynamic range neurons to mechanical noxious stimulation of a hindpaw were strongly inhibited by intravenous dipyrone (metamizol) [25].

Importantly, repeated microinjections of NSAIDs into the DH resulted in a progressive decrease in antinociceptive effectiveness, i.e. induced tolerance similar to that observed with intra-PAG, CeA and NRM injections [11,12,14-17], and reminiscent of the effect of opiates. For example, it has recently been shown that repeated intrathecal injections of a selective delta opioid receptor (DOPR) agonists deltorphin II or morphine induce tolerance effects on antihyperalgesic and antinociceptive responses in rodents [26].

A major involvement of opioidergic mechanisms in tolerance to the analgesic effect of NSAIDs is unusual, because traditionally the cellular and molecular actions of opioids were thought to differ from those of non-opioid analgesics. However, one interesting aspect of NSAIDs administration, namely tolerance, emphasizes their similarity to opioid analgesics. Indeed, microinjection of metamizol into PAG [13,15,16,18], or into CeA [11,18], progressively led to a loss of their antinociceptive effects, i.e. produced tolerance. Furthermore, tolerance to metamizol was accompanied by cross-tolerance to morphine as if opioid analgesics had been repeatedly administered [16,17].

The mechanism producing tolerance to NSAIDs can be due to the participation of endogenous opioids [19,27,28]. Herein we clearly showed that a non-selective opioid receptor antagonist naloxone significantly diminishes NSAIDs-induced antinociception on the first day, and on the second and third days shows trend effects. Our findings affirm the results of other investigators that microinjection of dipyrone and aspirin, and systemic dipyrone are abolished by systemically injected and/or microinjections of the opioid antagonists, naloxone, and CTOP (D-phe-Cys-Tyr-D-trp-Orn-thr-Pen-thr-NH2) [15,23,25,27]. The latter is a cyclic analog of the neuropeptide somatostatin and is known to block the analgesic effect of morphine [15]. Moreover, endogenous opioids are involved in the potentiation of analgesia observed with the combination of morphine plus dipyrone (metamizol). The release of endogenous opioids by dipyrone could enhance exogenous opiate effects, explaining the need for more naloxone to counteract the antinociception produced by morphine plus dipyrone [27].

The mechanisms whereby NSAIDs, in general, engage endogenous opioids are not completely understood. There is a proposed model for the PAG where *γ*-amino-butyric acid (GABA) containing synapses act as the plausible site where NSAIDs could converge with endogenous opioids. The PAG output neurons that drive antinociception via downstream relays, like the RVM, is tonically inhibited by local GABA-ergic synapses [19,28]. Endogenous opioids reduce presynaptic release of GABA in the PAG. Furthermore, activation of *μ*-opioid receptors in the PAG brings about an elevation of the intracellular concentration of arachidonic acid metabolites. One of the pathways leads to the formation of hepoxilins, which increase potassium conductance. This in turn hyperpolarizes the presynaptic GABA-ergic terminals and decreases GABA release [29]. Disinhibition of PAG output neurons would thus drive antinociception in the downstream loop of the PAG–RVM–spinal dorsal horn [30,31]. For this pathway to function, however, an activation of *μ*-opioid receptors seems to be necessary, because naloxone or CTOP blocks the effect of PAG-microinjected metamizol or aspirin [15,23].

As stated above, the action of either opioid or non-opioid analgesics in the PAG leads to an excitation of PAG output neurons and this causes an activation of RVM off-cells and an inhibition of RVM on-cells, thus leading to antinociception (analgesia). When tolerance develops, PAG microinjections of morphine [32], or metamizol [15], are no longer capable of affecting RVM neurons and inducing analgesia. These results show further neuronal relationships between opioid and non-opioid analgesics as regards the descending pain-control and modulation system [19,30]. In addition, metamizol probably can act via endocannabinoids in the downstream PAG-RVM axis reducing inflammation pain in rats [33].

There is evidence that GABAergic mediation of opioid effects is a widespread phenomenon and occurs throughout most of CNS. A co-localization between hippocampal *μ*-opioid receptors and GABA-ergic interneurons in CA1, CA3 and dentate gyrus has been shown in rats [34]. The localization of *μ*-opioid receptors on GABA-ergic neurons suggests that these receptors, when activated, can directly control the hippocampal GABA-ergic neurons’ activity [34,35]. Several studies have shown that activation of the opioid receptors can lead to the inhibition of interneuron activity resulting in diminished GABA release and the disinhibition of hippocampal pyramidal neurons [36-38].

Our results support the hypothesis that modulation of nociceptive response in the DH could occur in a manner similar to that proposed for the PAG [9,19,28,39]. It is therefore likely that in this study the antinociception observed after microinjection of NSAIDs into the DH occurs through the inhibition of tonically active GABA-ergic interneurons. In addition, involvement of the downstream PAG-RVM axis mechanism is also possible.

## Conclusions

In conclusion, our data showed for the first time that repeated microinjections of NSAIDs into the DH induce antinociception that is opioid mediated. These findings confirmed previous studies indicating that the antinociceptive action of NSAIDs may be mediated via the endogenous opioid system, as it is blocked by naloxone and exhibits tolerance.

## Competing interests

There is no conflict of interest in the present work.

## Authors’ contributions

GG, NT, and IN equally contributed to data collection. MN carried out histological control. MGT designed conception, finally analyzed the data and drafted the manuscript. All authors read, contributed to and approved the final manuscript.

## Pre-publication history

The pre-publication history for this paper can be accessed here:

http://www.biomedcentral.com/2050-6511/15/10/prepub
